# Global human-edible nutrient supplies, their sources, and correlations with agricultural environmental impact

**DOI:** 10.1038/s41598-022-21135-1

**Published:** 2022-10-06

**Authors:** R. R. White, C. B. Gleason

**Affiliations:** School of Animal Sciences, Virginia Tech, Blacksburg, VA 24061 USA

**Keywords:** Sustainability, Environmental impact

## Abstract

Food production, sustainable development, population growth, and agricultural environmental impacts are linked global problems that require complex solutions. Many efforts evaluating these challenges primarily evaluate dietary strategies designed for health and environmental objectives without considering the subsequent adaptations required by the global food supply. Here we use a complementary approach to summarize trends and variability in the current agricultural system in the context of the growing population and impending environmental challenges. Globally, agricultural systems produce sufficient nutrients to feed 10 billion people with the exception of Ca, DHA + EPA, vitamins B4, D, and E. In a network analysis, greenhouse gas emissions were conditionally dependent on ruminant meat and milk, while water use was conditionally dependent on vegetable and fruit production; however, supplies of most nutrients were also dependent on these same production categories, suggesting trade-offs between nutritional and environmental objectives. Future work should evaluate strategies to address these compromises (i.e., improving water use efficiency and reducing greenhouse gas emissions), to explore to what extent such compromises are biophysically essential or merely a product of the current agricultural system structures. Given the time-sensitive nature of population growth and environmental concerns, strategies to make more effective use of currently produced agricultural products will also be critical complementary strategies to sustainably feed the growing population which can work in concert with other agricultural-, diet- and policy-focused efforts.

## Introduction

In response to the apparent Malthusian crisis^1^ implied by the growing global population^2^, numerous investigations have sought to identify optimal human diets for global sustainability^3–5^. Such investigations are critically important because agricultural systems globally account for 76% of water withdrawal^6^ and 25% of greenhouse gas (GHG) emissions^6^. Furthermore, diet has a considerable effect on human health^7,8^, meaning changes in diet can be used to simultaneously address human health and environmental impact objectives. Because of the end-goal-driven nature of this problem, most existing investigations have focused on evaluating dietary strategies designed for health and reduced environmental impact objectives without considering the biophysical capacity of the agricultural system to support these diets. Although these assessments have the advantage of identifying out-of-the-box ideas and solutions, they can be prone to identifying infeasible options because they rely on what is theoretically possible, not what is physically (or practically) possible^9^. Complementing diet driven assessments with assessments of existent agricultural systems can provide a more comprehensive assessment of food systems options when attempting to progress toward sustainability. The objective of this study was to summarize current agricultural supplies of human-edible nutrients (HEN), their sources, and their correlation with agricultural GHG emissions and water withdrawal, in the context of the population at global, continent, and individual country scales. The goal of this summary process was to glean understanding of how current food systems contribute to nutrient supplies and environmental impacts through analysis of those systems which exist today.

## Results and discussion

### Sufficiency of macronutrient supplies and sources of supply

Agricultural supplies of nutrients produced after accounting for waste, trade, and animal feed use were stratified by nutrient and by food category within a continent and within individual countries (Supplementary Figs. S1 and S2). These total nutrient supplies were then compared with population nutrient requirements estimated from population age and gender distributions to evaluate nutrient sufficiency. Supplies were calculated using the P4 supplies described in the Supplementary Information (residual food available after accounting for trade, livestock feed, and waste). In a global assessment (Supplementary Fig. S3) the current agricultural system supplies sufficient human-edible nutrients to feed the current population with the exception of DHA + EPA and vitamin D. If the population were scaled to 10 billion people (i.e., an estimated future target population^10^), calcium, choline, and vitamin E would additionally be produced in insufficient supply. Comparison of the current agricultural system based on future population target requirements enables better conceptualization of how today’s system might be leveraged to feed a growing population, and is not intended to imply that the agricultural system will remain constant as the population grows and the climate changes over the next several decades. Because global analyses can mask local and geographically specific food limitations, we also analyzed continent- and country-specific food supplies in greater detail.

**Figure 1:**
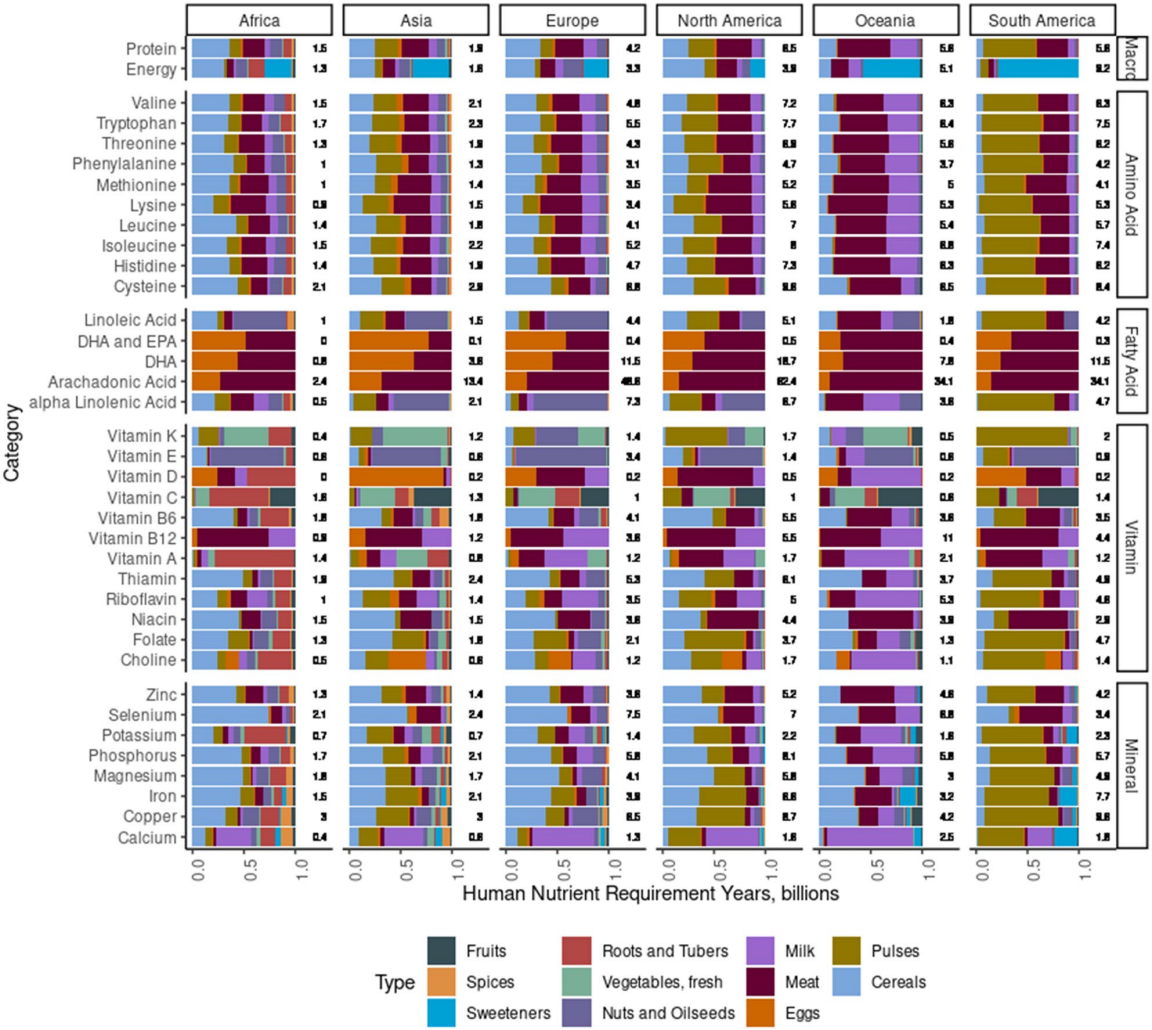
Breakdown of nutrient supplies by food type category and nutrient for each continent. Numbers reflect the supply of that nutrient expressed as a ratio of the population requirement for that nutrient.

Agricultural systems produced 1.3 to 8.2 times the energy necessary to meet requirements of the different continents’ populations (Fig. 1). Africa and Asia had the lowest levels of production relative to population requirements while North America, Oceania, and South America had the highest levels (Fig. 1). Despite being the lowest, agricultural systems in Africa and Asia produced 1.3 to 1.8 times the energy required by their populace. Regardless of this theoretical opportunity to exceed population energy needs, FAO data suggest more than 800 million people habitually under-consume energy globally^6^. The incongruencies between data on hungry populations in Africa and Southeast Asia, in particular^11^, and the energy production levels identified in this work provide some context to interpreting calculated supply numbers. A supply estimate exactly matching the population requirement implies adequate supply only if there are no: heterogenous socioeconomic distributions; habitual overconsumption by some individuals; underconsumption by others; challenges with food transport and distribution; and variation in true requirements among individuals.

**Figure 2:**
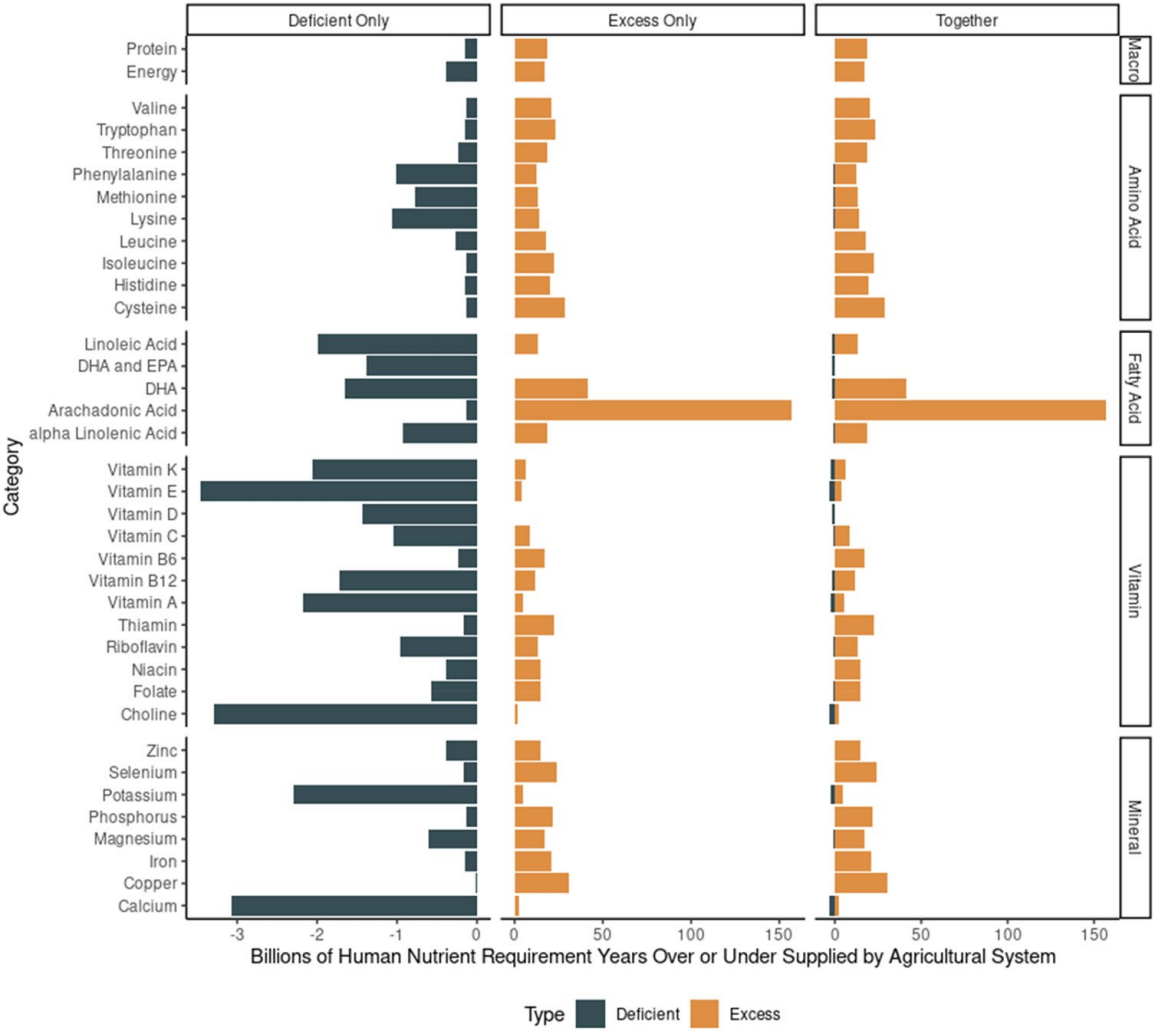
Comparison of nutrient provision (excess or deficient relative to population requirements) based on individual country data, summed to yield global supply estimates. Supply estimates used the P4 representation, which accounts for losses associated with waste and animal feed usage, along with changes in balance associated with trade. Values reflect the billions of human nutrient requirement years that are either over-supplied or under-supplied by the agricultural system, when supplies are scaled to the requirement of each country’s population. Nutrient deficiencies and excesses are shown on different panels for readability and on the same panel for scale comparison.

As might be expected from estimated global energy supplies (Supplementary Fig. S3), the nutrient requirement years oversupplied by agricultural systems producing sufficient energy far exceeds the deficiencies incurred in deficient countries (Fig. 2). Energy excesses are a separate human health concern because excess consumption of energy may lead to diabetes, obesity, and heart disease, among other ailments. The misbalance between countries producing excess energy supplies and those with energy deficiencies further supports equitable and accessible food distribution as a major challenge limiting our ability to feed the growing global population. Despite numerous countries with dramatic energy over-supply, the country-level shortage in agricultural supplies of energy suggests 390 million people are not provided sufficient energy by their domestic agricultural system (Fig. 2). When compared to the FAO estimate (800 million) of people habitually under-consuming energy, there is a clear disconnect between nutrients being supplied by the agricultural system and the consumption of nutrients by humans, further supporting the idea that coordination among the global agricultural systems could be a short-term strategy to address nutrient insufficiencies induced by limited domestic supplies. The fact that, under current energy production levels (over 4× population needs), approximately 10% of the global population habitually under-consumes energy suggests that strategies and policies which incentivize more efficient and equitable food use are essential and complementary approaches to agricultural systems interventions or dietary changes which have been previously proposed to address global food shortages. In addition to the findings yielded above, the individual country analysis could be employed as a strategy to discuss the concentration of nutrient scarcity issues (i.e., to examine those countries with deficiencies versus adequate supplies and identify if the supply of a particular scarce nutrient is diffuse or very concentrated). Across geographical locations, cereals and sweeteners provided the majority of energy available for consumption. Although cereals are often referred to as staple foods, sweeteners are referred to as “empty calories” because they provide energy without concurrently providing nutrients like protein, vitamins, and minerals; contribute to metabolic diseases; and disrupt regular hormone signaling^12^. Although the values are not intended to imply dietary consumption patterns and do not match the expectation based on the prevalence of sweeteners in the Western diet^13^, the supply estimates calculated herein suggest sweeteners supply 14 to 26% of the consumable energy supplied by agricultural systems in Northern America, Europe, and Africa; 34% of the consumable energy supplied in Asia; 54% of that supplied in Oceania, and over 78% of supplies in South America. The prevalence of sweeteners as an energy source may contribute to the pervasiveness of the obese but undernourished phenotype expressed throughout developed and developing societies^14^. Cereals in North America and sugars in South America are routinely used for ethanol production rather than human food consumption so further consideration of how industrial uses of agricultural products impact human food supplies is also important^15^.

Protein and amino acids produced by the agricultural production system ranged from 0.9 to 9.6 times that required for each continent’s population (Fig. 1). Cereals, meat, milk, and pulses were the major suppliers of protein (Fig. 1). Plant-based protein sources are often limited in concentrations of the essential amino acids Lys and Met^16^. Thus, it is not surprising that across all continents, Lys, Met, and Phe were produced in the lowest supplies. Those regions with higher proportion of protein coming from animal products (e.g., Oceania) had improved supplies of Lys and Met relative to those regions with limited supply coming from animal products (e.g., Africa and Asia). In Africa and Asia these amino acids were supplied in quantities close to sufficiency (0.9 to 1.5 times required), meaning that incomplete or heterogeneous distribution of these nutrients among food consumers will contribute to deficiencies. Data suggest stunting, a complex condition, can, among other things, infer insufficient intakes of adequate quality protein in children^17,18^. The prevalence of stunting in portions of Africa and Southeast Asia^19,20^, supports the idea that supplied protein quantity and quality in these regions, along with provision of other nutrients, should be strengthened. To further complicate the protein supply challenges in Africa and Asia, the comparison of protein supplies pre- and post-accounting for animal feed suggest that animal feeding systems in Africa and Asia are, on average, protein negative, implying that more protein is fed to livestock that is harvested from those animals. Because livestock products are an important source of nutrient-dense proteins and a complete amino acid source, addressing these inefficiencies in livestock production may be an important regional adaptation to support more sustainable food systems. As a final protein supply challenge, export from Asia and Africa indicate these regions are net protein importers, and retention of produced protein within these regions may better support societal demands for nutrient dense and high-quality amino acid sources. Collectively, the low supplies of protein and amino acids and the exacerbation of that challenge through inefficient protein feeding to livestock and protein export through trade highlights the need for focus on enhanced availability of amino-acid dense food categories, reconsideration of trade strategies, and enhanced efficiency of livestock feeding in these regions (Supplementary Fig. S2). As a complementary approach, protein sourcing from other regions of the world where protein is produced in dramatic excess may also support greater protein availability in Africa and Asia, though contributes to socioeconomic challenges such as concerns around nutritional imperialism.

### Sufficiency of micronutrient supplies and sources of supply

Individual studies have highlighted the role of lipids (i.e., EPA, DHA, ALA, and LA) in global disease burden^21^. Although some essential fatty acids are produced in ample supplies globally (e.g., arachidonic acid (AA)), others like EPA/DHA, linolenic acid, and linoleic acid have insufficient domestic production for 924 million to 1.98 billion people (Fig. 2). Meat and eggs made up the entirety of EPA, DHA, and AA sources globally (Fig. 1). Linoleic and alpha linolenic acids were supplied by a wider variety of foodstuffs with nuts, oilseeds and pulses being particularly important sources. The present assessment does not consider global supplies of seafood, which will undoubtedly contribute to consumable supplies of these essential fatty acids, particularly EPA and DHA.

Vitamins are among the more commonly noted deficiencies worldwide. In this investigation, vitamins A, B4 (choline), B12, C, D, E, and K were among the most insufficiently supplied nutrients (Fig. 2). The importance of different food categories in providing vitamin supplies differed by vitamin and by region (Fig. 1). For numerous B vitamins not commonly insufficiently supplied (i.e., B6, thiamin, riboflavin, niacin, and folate), cereals and pulses were important supplies. For those vitamins that were commonly insufficiently supplied, roots and tubers (vitamins A, E, and C); nuts and oilseeds (vitamins E and K); vegetables (vitamins A and K); pulses (vitamins B4 and K); milk (vitamins A, B4, B12, and D); meat (vitamins D and B12); and eggs (vitamins B4 and D) were important sources. The diversity in sources of vitamins highlights the necessary complexity of the agri-food system. Maintaining the diversity of agricultural products produced globally is important because there is no single food product or type that best sources all nutrients provided in least supply, which suggests that human diets require a variety of different food types because of the complementary nutrient profiles provided.

Of the vitamins in low supply, vitamin D supplies were insufficient for 1.43 billion people on a country-level (Fig. 2). This severity of insufficiency matches claims for the high prevalence of vitamin D deficiency worldwide^22,23^. Choline supplies were insufficient for 3.28 billion people (Fig. 2), which was surprising because global choline insufficiency is not a subject widely discussed within the scientific literature. Further investigation is warranted on why choline deficiencies are not more broadly identified, given the apparently low supplies globally. Conversely, vitamins A^24^, E^25^, and K^26^ have previously been identified as frequently deficient in populations around the world, thus their low estimated supplies was consistent with previous literature. A summary of surveys on vitamin B12 suggests 5 to 35% of the global population experiences deficiencies in B12^27^, within the present work 1.7 billion people were estimated to have insufficient domestic vitamin B12 supplied by their countries’ agricultural system. Although excesses of most of these vitamins exceed the deficiencies, choline, vitamin A, and D were close to balanced between estimated deficiencies and excesses, meaning increased global supplies of these nutrients should be a priority because socioeconomic pressures causing heterogeneity in food supply distribution will contribute to insufficient supplies in many regions.

The most commonly observed mineral deficiencies globally are iron, iodine, and zinc^28^. In deficient countries, the agricultural system failed to provide Fe to meet the needs of 149 million people and Zn to meet the needs of 393 million (Fig. 2). Interestingly, these two minerals were supplied in greater quantities than many others assessed. Potassium and calcium were the most insufficiently supplied minerals (Fig. 2) with 2.29 and 3.07 billion people’s requirements not being supplied by their domestic agricultural systems. Calcium insufficiency has been highlighted globally, typically in discussions of nutritional rickets and in association with limitations in vitamin D^29^. A study on the prevalence of low Ca intakes worldwide also found limited Ca consumption in most countries; however, these authors highlighted the need for more comprehensive data to better understand the severity of limitations in Ca intake^30^. Milk and pulses were the most important sources of Ca across each continent (Fig. 1). Adapting agricultural systems to provide improved supplies of Ca, supplementing, or fortifying foods to enhance Ca availability have been widely discussed as solutions to the low global provision of Ca and should collectively be considered as strategies to address this challenge (Supplementary Fig. 3). In contrast, K deficiency has not been widely reported and should be evaluated further. Sources of K were varied and included roots and tubers (Africa), pulses and cereals (Asia, Europe, North America, South America), and milk (Oceania; Fig. 1).

Previous studies have identified physiological differences in nutrient and energy metabolism, retention, and use among different demographic groups^31–33^. Within the present work, the nutrient targets were based on age and gender differences only and did not reflect any known or presumed differences among racial/ethnic groups, disease states, climate influences, etc. Similarly, nutrient targets did not account for any known nutrient interactions, and regional/varietal differences in crop and animal nutrient compositions were not considered. More precise accounting for some of these regionally specific factors may shift the profile of nutrients supplied and required within individual countries. Because comprehensive datasets on these more regionally specific variations are not presently available, it was not feasible to include the expected variability within the present analysis.

### Environmental impacts and nutrient supplies

**Figure 3:**
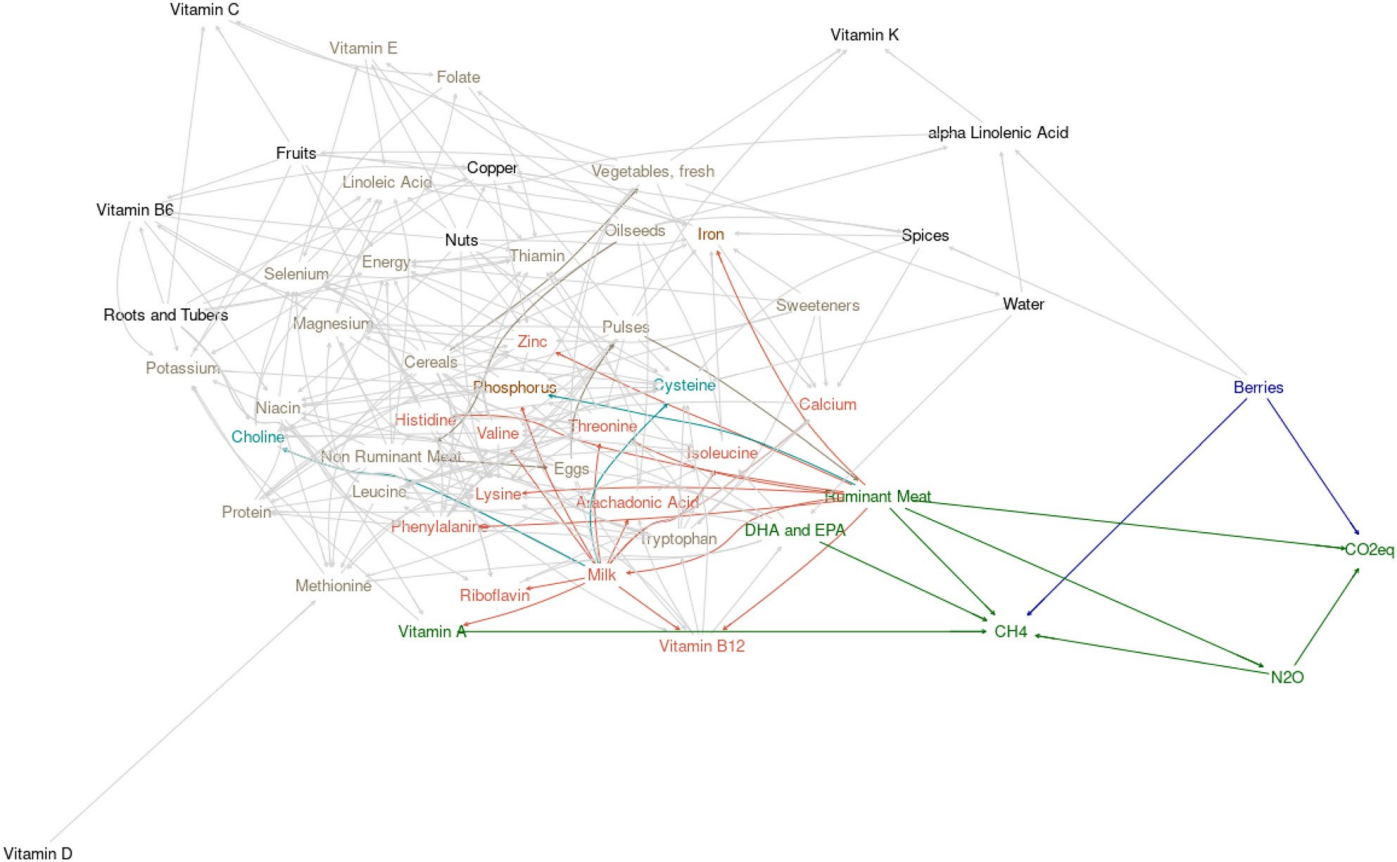
Bayesian learning network relating individual country yearly average production of different categories of foods (total mass produced), production of different nutrients and energy (total amount produced), and agricultural production of methane (CH_4_), nitrous oxide (N_2_O), and carbon dioxide equivalent (CO_2_eq), and agricultural water use. Nodes are colored to highlight the relationships revealed by exploring the Markov blankets of CO_2_eq, CH_4_ and N_2_O. Navy nodes and edges show indirect conditional dependencies driving CO_2_eq, CH_4_ and N_2_O. Green nodes and edges show direct conditional dependencies driving CO_2_eq, CH_4_ and N_2_O. Red nodes reflect nutrients expected to decrease if ruminant meat was targeted as a strategy to decrease greenhouse gas emissions. Light blue nodes reflect those expected to increase if ruminant meat was targeted as a strategy to decrease greenhouse gas emissions. The variables highlighted in the tan colors show the broader nutrient blanket expected to be conditionally dependent on ruminant meat and milk production but through more than 1 degree of separation.

**Figure 4:**
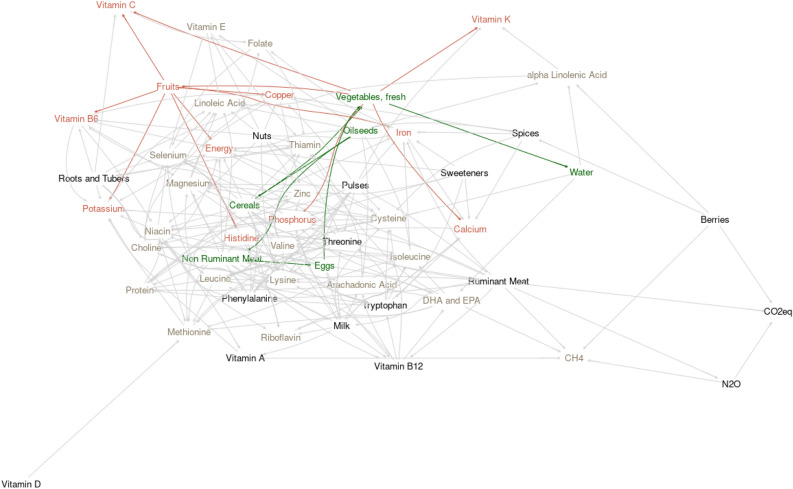
Bayesian learning network relating individual country yearly average production of different categories of foods (total mass produced), production of different nutrients and energy (total amount produced), and agricultural production of methane (CH_4_), nitrous oxide (N_2_O), and carbon dioxide equivalent (CO_2_eq), and agricultural water use. Nodes are colored to highlight the relationships revealed by exploring the Markov blanket of agricultural water use. Green nodes and edges show conditional dependencies with direct associations driving agricultural water use. Red colored nodes and edges show negative collateral impacts associated with targeting vegetable and fruit production as a strategy for reducing agricultural water use. The variables highlighted in the tan colors show the broader nutrient blanket expected to be conditionally dependent on vegetable and fruit production but through more than 1 degree of separation.

Figures 3 and 4 depict a Bayesian learning network relating country-level supplies of foods and nutrients, agricultural GHG emissions, and agricultural water withdrawal, each scaled per capita to avoid skewing by large population countries. Figure 3 highlights the nodes and edges within the network which are associated with targeting strategies to lower per capita CH_4_, N_2_O, or CO_2_ equivalents, while Fig. 4 highlights the nodes and edges within the network which are associated with targeting strategies to lower per capita water use. Aside coloring to display key relationships, the networks underlying each figure are identical. The words within each image reflect nodes (i.e., variables) within the network, and arrows reflect conditional dependencies. Arrows are directed implying the node pointed to is conditionally dependent on the node where the arrow originates. Those conditional dependencies also have directionality in terms of the type of association (i.e., inverse versus direct), which are largely codified by the color patterns in each figure. Identified linear relationships and standard errors of associations within the network are presented (Supplementary Table S1).

The network revealed a driving role of oilseed production in agricultural systems, highlighted by a cascade of conditional dependencies and positive linear associations where oilseed production drove (directly or indirectly), production of cereals, eggs, sweeteners, non-ruminant meat, vegetables, fruits, pulses, ruminant meat, and milk. Production of nuts, roots and tubers, and berries were largely independent of the oilseed-driven cascade of relationships associating those more prevalent and traditional agricultural products. This was sensible because many industrialized agricultural systems tend to rely on cash crops and production of livestock both to supply domestic demand and to support economically viable trade. Nuts, roots and tubers, and berries require more specialized environmental conditions and are likely more driven by climate than by other agricultural system factors.

Assessment of greenhouse gas nodes revealed conditional dependencies on berries and ruminant meat. Methane was additionally conditionally dependent on DHA + EPA and vitamin A supplies within systems. Associations among greenhouse gas variables and berries were inverse, suggesting those systems with greater berry production per capita tended to have lower greenhouse gas emissions. The other associations were direct, suggesting that with greater production of ruminant meat, and provision of DHA + EPA and vitamin A came greater production of greenhouse gases. Ruminant meat was highly central within the network and was tightly linked with milk production. To explore the impacts of reducing ruminant meat and milk production as a strategy to control greenhouse gas emissions, we explored the directionality of conditional dependencies relating nutrient provision to supplies of meat. Twelve nutrients were directly associated with ruminant meat and milk production, while two (choline and cysteine) were indirectly associated. Ruminant meat production was involved in the cascade of agricultural products driven by oilseeds and the broader implications of shifting relationships upstream in that production cascade revealed that only 5 nutrients (vitamin B6, vitamin C, vitamin K, copper, and alpha linolenic acid) were conditionally independent of ruminant meat. As such, the assessment suggests that production of ruminant animals within food systems represents a biophysical compromise within the food system whereby provision of low-supply vitamins, minerals, amino acids, and fatty acids is improved but at the expense of elevated greenhouse gas emissions. As such, direct mitigation of greenhouse gas emissions from ruminant agriculture may be a strategy to address this compromise without negatively influencing nutrient profiles.

Agricultural water use was conditionally dependent and directly associated with production of vegetables. Much like ruminant meat and milk, fruits and vegetables were strongly linked with fruits revealed as conditionally dependent upon and directly associated with vegetable production at the country level. Water use was conditionally independent of greenhouse gas emissions variables, revealing different strategies may be needed to address different aspects of agricultural environmental impacts. Much like was observed with targeting ruminant meat as a strategy to reduce water use, a number of nutrient supplies were conditionally dependent on production levels of fruits and vegetables, thus reducing production of fruits and vegetables may have unintended negative consequences on supplies of energy, potassium, copper, phosphorus, histidine, calcium, and vitamins K, C, and B6. Similarly, fruits and vegetables were implicated in the cascade of production variables driven by oilseed production, and the broader nutrients which may be influenced by shifts in that pathway driving vegetable production influenced all nutrients except phenylalanine, threonine, tryptophan, and vitamin B12. Collectively, both analyses suggest that nutrients, broadly, are conditionally dependent on production choices within the agricultural systems. Much like those strategies suggested by the analysis on GHG emissions, improving the water use efficiency of fruits and vegetables may be a compromise to support enhanced environmental impacts of production systems and provision of adequate nutrients for populations.

## Conclusions

Fully recognizing that the comparison of humanity’s environmental impact with respect to proposed planetary boundaries^34^ highlights the need for rapid responses to environmental challenges, the data presented herein suggest that considerable nutrient excesses are currently generated by agricultural systems globally. The excesses indicate the idea that we must produce more food to support a growing global population is an oversimplification and somewhat inaccurate. Alternative priorities that should be considered for globally-focused efforts toward building more sustainable food supply systems include: (1) promoting coordination within the global agricultural system to facilitate improved affordability and accessibility of produced foods; (2) improving cooperation among food system sectors to highlight the essential aspects and complementarity of all food types; and (3) characterizing the extent to which compromises among nutrient supplies, GHG emissions, and water withdrawal can be tolerated within the global context. Although it must be an objective of all sectors to try and reduce environmental impacts, the opportunities to do so while continuing to enhance available supplies of domestically or globally limiting nutrients will undoubtedly require more individualized efforts (e.g., enhancing protein-use efficiency of livestock production and trade systems in Asia and Africa), and the complexity of country-specific analyses supports the idea that geographic specificity is essential in considering appropriate supply-side interventions supporting food sustainability.

## Methods

### Data overview

To conduct this analysis, we sourced 10 years of agricultural production data from FAOSTAT^6^, mapped agricultural products to foods, and sourced composition of those foods from the USDA food nutrient composition database^35^ to estimate supplies of nutrients produced within the agricultural sector. Livestock production data and crop production data were both sourced from FAOSTAT^6^. Food import, export, and waste data from FAO^6^ were also leveraged to account for food transportation and wastes. Food consumption by livestock was estimated from the region-specific breakdown in Mottet et al.^36^. Different supply representations were calculated to contextualize the relative importance of these different food fluxes in the global food landscape. These supplies were compared to human nutrient requirements calculated based on age- and gender-specific requirements^37,38^ and age- and gender-based population distributions^10^. Greenhouse gas emissions estimated by FAO^6^ were also sourced to understand correlations among GHG, nutrient supplies, and agricultural systems.

### Data sources and cross-referencing

Agricultural production data was sourced from two downloaded datasets obtained from FAOSTAT^6^. The crop production data included 168 primary crop products from 240 geographical areas from 2008 to 2017. These geographical regions reflected the FAO calculated values for global and continent data, as well as reported values for individual countries. The animal production data included 44 primary products from the same geographical areas. Of these 212 primary products, composition data were sourced from USDA^35^ for 206. The products without nutrient composition information included fibrous products that are not used for human food and tobacco and related drugs that are chewed or smoked rather than consumed for nutrients. Because the nutrient composition and food supply data were from different sources, a cross-reference was developed to map the name in the FAO database to the associated name in the USDA database. This cross-reference was merged into each dataset and used for cross-referencing when merging and making calculations from these datasets.

Datasets estimating populations of men and women in different age groups in 255 geographical regions throughout the world were downloaded for reference dates of 2005, 2010, 2015, and 2020^10^. The age groups reflected 5-year intervals from birth (0 years to 99 years). Individuals greater than 100 years of age were grouped into a single category. Because the timespans did not match perfectly with the FAO data, in cross-referencing between this dataset and the yearly FAO data, 2005 data were used for 2008, 2010 data were used for 2009 through 2013, 2015 data were used for 2014 through 2016 and 2020 data were used for 2017. The datasets also used different names to refer to similar geographical locations (often spelling the names of countries differently or ordering words differently within a country name) and a cross-reference was created to map from the FAO areas list to the UN areas list.

A requirement dataset was developed based on the age- and gender-specific requirements^37,38^ to match the age groupings provided by the UN population database. The names of the different populations were then used to cross-reference the requirement database to the population database for calculating weighted average requirements for a population within a given time range.

Because of inconsistencies and incompleteness of the FAO food loss and waste database for specific countries and food products, food waste aggregate estimates for regions and food types were used to estimate food loss and waste^39^. Loss, which included production, postharvest, and processing losses, was differentiated from waste and was assumed to occur prior to accounting for the import and export of goods, waste was assumed to occur after trade of goods. Cross-references were developed to map food categories to individual food products and regions to individual countries (where necessary).

Trade data (import and export) from FAO^6^ were leveraged to estimate the quantity of agricultural products being imported and exported annually by different geographical regions. Although the trade data were from the same source, minor technical editing (missing commas, parentheses, altered spelling, etc.) of some food product descriptions were needed to cross reference between trade data and production data.

Environmental impact datasets were referenced from FAO calculated values^6^ and downloaded for the geographical regions of interest between 2008 and 2017. Because of the coherence with the other FAO data used in the analysis, no specific cross-references were needed to integrate these data with production data.

Animal feed intake data were estimated based on representative animal diets from OECD countries and from non-OECD countries based on different production styles^36^. Daily animal intakes were differentiated to reflect ruminant and non-ruminant diets. The relative proportions of animals housed under each production style were calculated based on the values presented in Mottet et al.^36^. These proportions were then merged into the animal dietary dataset and used to calculate a weighted average diet for each animal type. The animal types were then cross-referenced to 10 years of region-specific animal population estimates from FAO^6^ and used to calculate total intake of different food categories based on animal type each year. Because the geographical regions used in this paper did not match the FAO geographical regions perfectly, a separate regional cross-reference was created to map between these datasets.

### Calculating supplies of food

Four different food supplies were calculated. These are reflected in the four representations shown in the different figures. Representations included: P1, agricultural production without animal products; P2, P1 with food losses removed and then trade accounted for; P3, P2 with food waste accounted for; and P4, P3 with animal products and animal feed consumption accounted for. To calculate these different supplies, the crop and animal production data were merged into a single agricultural production dataset. The cross-references in product name and location were then used to merge in estimates of the proportion of each product lost to (or provided by) each food flux. Because of incomplete reporting of regions across the datasets, the resulting merged production data contained information for 222 unique geographical regions and 213 agricultural products. Missing values (e.g., missing production estimates for a product in a country) were assumed to be 0. The total kg of each food produced was calculated as 1000 times the metric tonnes of food reported to be produced in the production data set. The same conversion was used to calculate the kg of food imported and exported from each region. The net effect of trade was calculated as the difference between production after accounting for food losses plus imports, minus exports. If this value was less than 0, it was assumed to be 0 because total exports from a country were assumed to be bounded by domestic production plus imports. The amount of food wasted was estimated by multiplying the food supply after accounting for trade by the proportion of food estimated to be wasted. The amount of food going to livestock feed was calculated as the food supply after accounting for trade multiplied by the proportion of individual feeds estimated as fed to livestock. The amount of food coming from livestock sources was calculated by summing food from meat, milk, and eggs. The different supply representations were then calculated based on these estimated food fluxes. The P1 supply was calculated as the raw production quantities without livestock products. The P2 supplies were those obtained from P1 after subtracting losses and adding the net contribution of trade. The P3 supplies were estimated as the P2 supplies minus the fluxes associated with food waste. The P4 supplies were estimated as the P3 supplies plus the production quantities of livestock products, minus the flux of fed feed. These values were averaged over the available years of data for reporting in the manuscript.

### Calculating human nutrient requirements and ratios of supply to requirements

Human nutrient requirements were calculated much the same as described in our previous work^40^ but with different age groups considered. The population database was cross-referenced to the requirements database and used to generate an estimate of the requirement of a geographical area for a year. That requirement was divided by 365 to generate daily requirements, which were compared to the original requirement databases as a sense check. These requirements were then averaged over the available years of data to yield the requirements used for referencing throughout the work. The different supply representations described above were cross-referenced to the annual requirement of each geographical region to determine the sufficiency of the nutrients supplied by the agricultural system.

### Examining relationships among supplies and impacts

To explore the relationships among environmental impacts and agricultural supplies, the individual country level data were used. The GHG emissions database, the water withdrawal database, and the production database were merged into a single database with one row for each country. A Bayesian learning network^41,42^ was then developed to examine the relationships between individual country yearly average production of different categories of foods (based on the total mass produced), production of different nutrients and energy (based on the total amount produced), and agricultural production of CH_4_, N_2_O, and CO_2_ equivalents (CO_2_eq), and agricultural water use. Bayesian learning networks allow for examination of structural associations among complex datasets by highlighting their conditional dependencies in a directed acyclic graph. This network derivation strategy was well suited to analysis of the associations among food production, nutrient supplies, and environmental impacts because the strategy can incorporate expected knowledge, highlight conditionally dependent and independent relationships within complex datasets, and provide a structured, graphical relationship of the probabilistic relationships identified. All values were scaled per capita to better control for mass variations associated with country population sizes. Expert knowledge was incorporated into the network structure by blacklisting associations from nutrient supplies to food categories (i.e., milk production could not be conditionally dependent on calcium supplies, but the opposite could be true). A score-based hill-climbing algorithm was used for structure learning. Arc strength and directional probabilities were calculated based on model averaging across 200 bootstrapped derivations. A parsimonious network was selected by retaining arcs with strength greater than 0.6 based on graphical evaluation of the conditional density function of arc strengths versus the minim required arc strength. The resulting network was fully directed with 54 notes, 250 edges, and an average Markov blanket size of 22.7 nodes. The network was visualized with the Rgraphviz package^43^, and used to explore structural relationships revealed by the conditional dependent of CO_2_eq on ruminant meat production and the conditional dependence of water use on vegetable production.

## Supplementary Information

Supplementary Information.

## Data Availability

The datasets used in the analysis are available on the open-access Virginia Tech Data Repository (10.7294/6y9v-gg39). The code used to generate figures presented in the analysis is archived at https://github.com/rrwhitevt/Global-Agricultural-Supplies-of-Human-Edible-Nutrients-Their-Sources-And-Their-Correlation-with-Ag/tree/master.
