# Supplementary material for: Global human-edible nutrient supplies, their sources, and correlations with agricultural environmental impact
Source: Sci Rep. 2022 Oct 6;12:16781. doi: 10.1038/s41598-022-21135-1 (PMC9537515; doi:10.1038/s41598-022-21135-1)
Supplement: Supplementary file 1 — Supplementary Information. [file 41598_2022_21135_MOESM1_ESM.docx]

**Supplementary Information**

Agricultural systems are complex, adaptive, and comprised of numerous individual heterogenous components that drive system behaviors through independent and interacting influences^1^. In current agricultural systems, there are at least 5 food fluxes of interest with respect to feeding the growing global population including food: production, exports, imports, waste, and use for livestock feed. Food loss was differentiated from waste and was assumed to occur prior to accounting for import and export of goods. The difference between domestic food production plus imports minus exports, waste, and feed should yield a reference supply of food (termed here as “residual”) potentially available for domestic use. It is important to note that this residual is very different from human consumption and should not be considered a proxy for consumption in any way. Using FAO production, trade, and waste data^2^, and estimates of animal feed consumption by food type and region^3^, we estimated the weight of food products associated with these different fluxes on each continent (Fig. S1).

The importance of different food fluxes differs by food type (Fig. S1). For all food types, the residual food supply (production + imports – waste – feed – exports) averaged the largest food flux across categories, meaning that, on average, the majority of agricultural supplies are available for use for food, industrial processes, and other uses. Food waste averaged the second-largest flux and was particularly impactful on the residual supplies of vegetables, roots and tubers, and fruits and berries. The wastage of fruits and vegetables were in the greatest proportions in Africa, Asia, and South America. Roots and tubers were wasted in greater proportion in Northern America, Europe, and Oceania.

Imports and exports were important food fluxes for select food types. Spices were exported from Africa, Oceania, and South America and imported by Europe, North America, and Oceania. Pulses, nuts, and oilseeds were exported from North America, Oceania, and South America and were imported by Africa, Asia, and Europe. There was also some international transit of fruits, vegetables, cereals, and animal products, but to a much lower extent.

Use of food for animal feed averaged the smallest food flux and was most important for influencing residual quantities of pulses and cereals. When assessed at the global scale, 1.22 billion T of food was provided to animals and 2.90 billion T was wasted by humans within the food system. Exports averaged 0.725 billion T. Residual food supplies totaled 5.00 billion T. These values suggest that eliminating food wastage could expand food availability by 58%. Removing ingredients from livestock feed that compete with human food production would result in a 24% expansion in food availability, assuming no change in livestock product outputs on this non-competitive diet, which may be inaccurate.

To better account for how different uses of agricultural supplies impact the nutrient profile of consumable food, we tracked how the supplies of 37 nutrients change between representations accounting for basal agricultural production with no livestock products (P1); P1 plus imports less exports (P2); P2 minus waste (P3); and P3 plus nutrients from animal-source foods minus fed nutrients (P4). For each continent (Fig. S2) and at the global scale (Fig. S3) the supplies of nutrients under these different representations were expressed relative to the population’s requirement for each nutrient to provide context for supply numbers. A calculated average requirement for the continent or global population was based on the age and gender demographics of each geographical area. The supplies were ratioed to the calculated requirements to determine where food supplies failed to meet requirements. For the global analyses we also determined which additional nutrients would be produced in insufficient supply if feeding a population of 10 billion (Fig. S3).

Across nutrients, food waste (demonstrated as the difference between P3 and P2) decreased nutrient provision (Fig. S2). The effects of trade and livestock varied by location and nutrient (Fig. S2). Trade, exemplified as the difference between P1 and P2, improved supplies of proteins and amino acids in Africa and Asia, but not in North America, Oceania, and South America (Fig. S2). This shift in nutrient availability suggests trade is already being leveraged as a short-term strategy to improve provision of nutrients available in regions with numerous deficiencies such as Africa and Asia while efforts to establish food sovereignty are being enacted.

Livestock farming, shown as the difference between P3 and P4, yielded improvements in nearly all nutrient supplies in North America, Oceania, South America, and Europe (Fig. S2). Livestock farming in Africa and Asia did not always result in net improvements in nutrient supplies, particularly for amino acids, some vitamins, and minerals (Fig. S2). The fact that livestock farming did not improve production of nutrients in these nutrition-insecure areas highlights major challenges with the efficiency of livestock farming in these regions that must be addressed for enhanced food sovereignty. These data also highlight that country-specific investigations (e.g., White and Hall^4^) should be considered only valid for the country from which the data were sourced.

Globally, the food system (P4 representation) produces sufficient supplies of human-edible nutrients to meet the needs of a 10-billion-person population with the exception of DHA+EPA, vitamins B4 (choline), D, and E, and calcium (Fig. S3). The excess global provision of most nutrients is not addressed in many previous studies focused on human diets, which suggests we must expand the agricultural system to meet human needs^5–7^. By contrast, the data presented herein suggest a static (or even reduced) agricultural system which, if reapportioned to enhance and direct supplies of limiting nutrients, could serve to feed the growing population. Importantly, the conclusion rests on the improbable and challenging caveat that the socio-eco-political and practical barriers to food trade and food transportation/storage could be adapted to ensure food excesses are transported with high precision to areas with food insufficiencies. However, the data importantly bring to light the opportunity to enhance coordination within the global agricultural system as an alternative to dramatic dietary or agricultural production shifts which may or may not be feasible or appropriate.

**
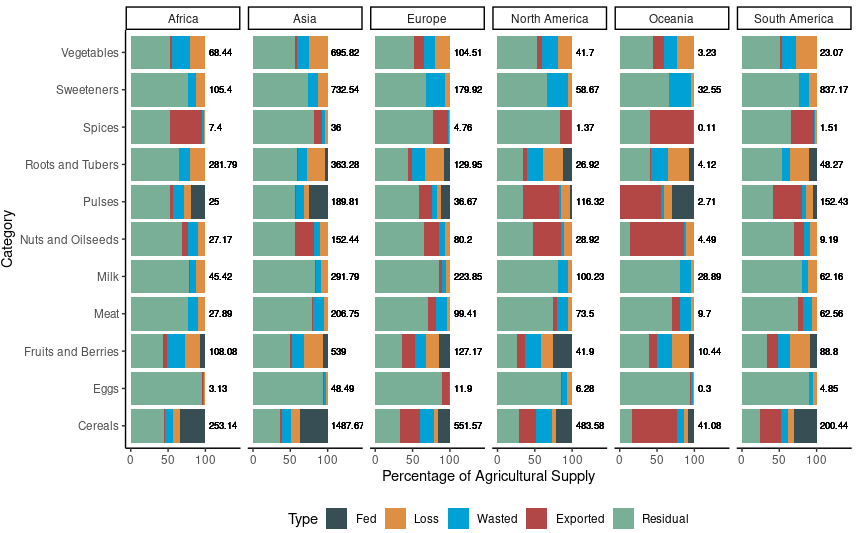
**

Figure S1. Mass of foods exported, wasted, lost, used for livestock feed, and remaining for domestic use by food type and continent. Fed reflects the mass of human-edible foods fed to livestock. Exported indicates the masses of foods exported. Wasted indicates the estimated mass of food wasted. Residual indicates the difference between agricultural supply plus imports less exports, waste, and animal feeding fluxes. Loss was assumed to occur prior to accounting for import and export of goods. Values are expressed as a percentage of the total and the numbers next to each bar are the millions of metric tonnes within the system.


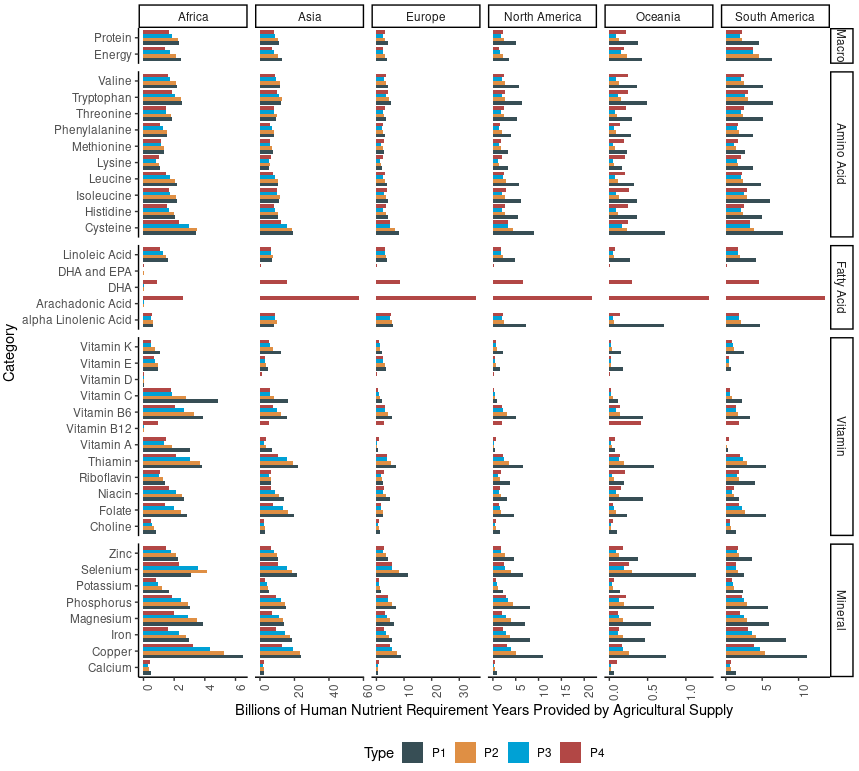


Figure S2. Breakdown of supply of nutrients from different representation considered. Representations included: P1, agricultural production without animal products; P2, P1 with trade accounted for; P3, P2 with waste accounted for; and P4, P3 with animal products and animal feed consumption accounted for. Supplies are expressed as a ratio of population requirements, meaning a value of 1 will provide sufficient supply to exactly meet the requirement of the population.


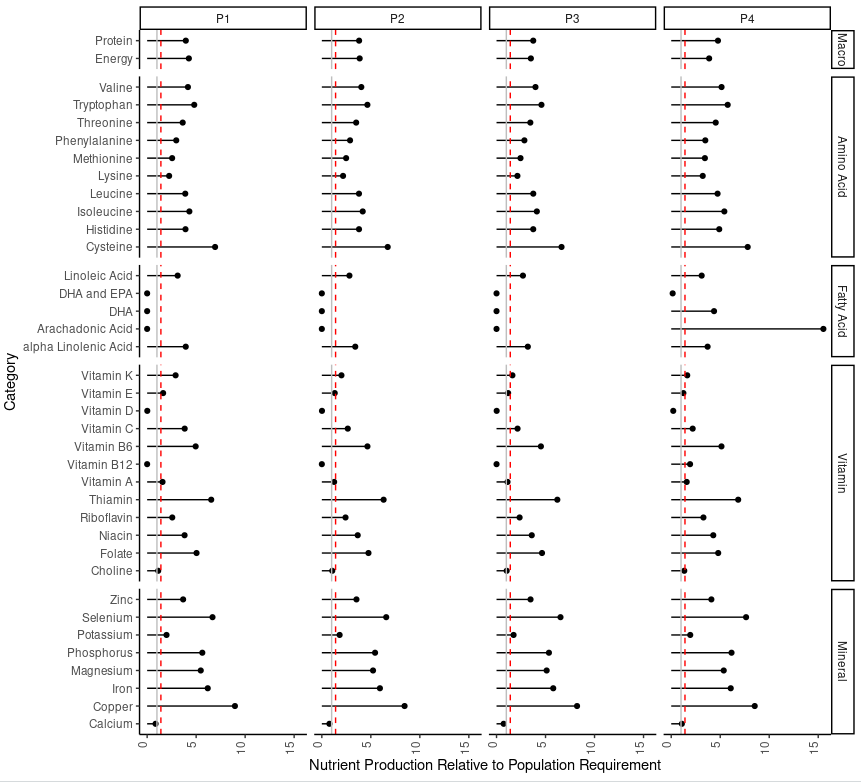


Figure S3. Global quantities of nutrients produced under each calculation type, with reference lines showing that needed to feed the current population (grey) and the quantity needed to feed a population of 10 billion (red dashed). Representations included: P1, agricultural production without animal products; P2, P1 with trade accounted for; P3, P2 with waste accounted for; and P4, P3 with animal products and animal feed consumption accounted for. In the case of global nutrient supplies, we would expect P1 and P2 to be identical because trade should not create new supplies of food.

**Supplementary References**

1. Nesheim, M. C. *et al.* A Framework for Assessing the Food System and Its Effects. in *A Framework for Assessing Effects of the Food System* (National Academies Press (US), 2015).

2. FAO. FAOSTAT. http://www.fao.org/faostat/en/#data/QA (2019).

3. Mottet, A. *et al.* Livestock: On our plates or eating at our table? A new analysis of the feed/food debate. *Glob. Food Secur.* **14**, 1–8 (2017).

4. White, R. R. & Hall, M. B. Nutritional and greenhouse gas impacts of removing animals from US agriculture. *Proc. Natl. Acad. Sci.* **114**, E10301–E10308 (2017).

5. Delgado, C. L. Rising consumption of meat and milk in developing countries has created a new food revolution. *J. Nutr.* **133**, 3907S-3910S (2003).

6. Conway, G. & Toenniessen, G. Feeding the world in the twenty-first century. *Nature* **402**, C55–C58 (1999).

7. Godfray, H. C. J. *et al.* Food security: The challenge of feeding 9 billion people. *Science* **327**, 812–818 (2010).

**Supplementary Table**

**Supplementary Table S1.** Identified linear relationships and standard errors of associations within the Bayesian learning network

| Response | Intercept | Variable | Value | Strength | Direction | Residual SE |
| --- | --- | --- | --- | --- | --- | --- |
| Energy | 0.00938 | Cereals | 0.0022 | 0.99 | 1.0 | 0.0722 (2.9%) |
|  |  | Sweeteners | 0.0052 | 1.0 | 1.0 |  |
|  |  | Oilseeds | 0.010 | 0.99 | 1.0 |  |
|  |  | Fruits | 0.00096 | 0.99 | 1.0 |  |
|  |  | Roots & Tubers | 0.0011 | 0.90 | 1.0 |  |
|  |  | Non-Ruminant Meat | 0.0029 | 0.87 | 1.0 |  |
|  |  | Cysteine | -0.202 | 0.63 | 0.98 |  |
|  |  | Vitamin B6 | -0.114 | 0.81 | 0.99 |  |
|  |  | Thiamin | 0.172 | 0.85 | 0.96 |  |
|  |  | Choline | 0.402 | 0.67 | 0.94 |  |
|  |  | Zinc | 0.494 | 0.70 | 0.91 |  |
|  |  |  |  |  |  |  |
| Protein | 0.183 | Roots & Tubers | 0.0035 | 0.64 | 1.0 | 0.168 (6.0%) |
|  |  | Non-Ruminant Meat | 0.0023 | 0.63 | 1.0 |  |
|  |  | Threonine | 0.0786 | 0.66 | 0.57 |  |
|  |  | Phenylalanine | 1.16 | 0.90 | 0.68 |  |
|  |  | Arachidonic Acid | -0.0013 | 0.68 | 0.97 |  |
|  |  |  |  |  |  |  |
| Cysteine | 0.0042 | Cereals | 0.0022 | 0.80 | 1.0 | 0.100 (2.4%) |
|  |  | Pulses | -0.0054 | 0.79 | 1.0 |  |
|  |  | Spices | -0.0087 | 0.73 | 1.0 |  |
|  |  | Milk | -0.0019 | 0.81 | 1.0 |  |
|  |  | Tryptophan | 0.29 | 0.88 | 0.94 |  |
|  |  | Phenylalanine | 1.59 | 0.74 | 0.88 |  |
|  |  | Leucine | -0.84 | 0.99 | 0.72 |  |
|  |  | Isoleucine | 0.55 | 0.62 | 0.80 |  |
|  |  | Vitamin B12 | -0.062 | 0.82 | 0.96 |  |
|  |  | Phosphorus | 0.21 | 0.68 | 0.51 |  |
|  |  | Water | 61,617 | 0.67 | 1.0 |  |
|  |  |  |  |  |  |  |
| Histidine | -0.20 | Fruits | 0.0018 | 0.85 | 1.0 | 0.112 (3.8%) |
|  |  | Protein | 0.61 | 0.82 | 0.68 |  |
|  |  | Valine | 0.62 | 0.63 | 0.82 |  |
|  |  | Tryptophan | -0.22 | 0.89 | 0.81 |  |
|  |  | Threonine | 0.53 | 0.79 | 0.85 |  |
|  |  | Phenylalanine | -0.88 | 0.93 | 0.79 |  |
|  |  | Cysteine | 0.14 | 0.73 | 0.57 |  |
|  |  |  |  |  |  |  |
| Isoleucine | -0.027 | Tryptophan | 0.056 | 0.91 | 0.61 | 0.092 (2.7%) |
|  |  | Threonine | 1.14 | 0.97 | 0.98 |  |
|  |  | DHA+EPA | 0.42 | 0.63 | 0.94 |  |
|  |  | Arachidonic Acid | -0.0029 | 0.64 | 0.95 |  |
|  |  |  |  |  |  | *(Continued)* |
| **Supplementary Table S1.** Continued | | |  |  |  |  |
| Response | Intercept | Variable | Value | Strength | Direction | Residual SE |
| Leucine | -0.057 | Cereals | 0.00043 | 0.77 | 1.0 |  |
|  |  | Protein | 0.28 | 0.86 | 0.65 |  |
|  |  | Tryptophan | -0.48 | 0.86 | 0.67 |  |
|  |  | Threonine | 0.19 | 0.74 | 0.71 |  |
|  |  | Phenylalanine | 0.76 | 0.71 | 0.62 |  |
|  |  | Isoleucine | 0.47 | 0.69 | 0.58 |  |
|  |  |  |  |  |  |  |
| Lysine | -0.010 | Cereals | -0.0012 | 0.99 | 1.0 | 0.019 (0.9%) |
|  |  | Pulses | 0.00095 | 0.77 | 1.0 |  |
|  |  | Nuts | -0.0028 | 0.98 | 1.0 |  |
|  |  | Ruminant Meat | 0.00044 | 0.78 | 1.0 |  |
|  |  | Non-Ruminant Meat | 0.0016 | 0.95 | 1.0 |  |
|  |  | Valine | 0.012 | 0.65 | 0.78 |  |
|  |  | Threonine | 0.063 | 1.0 | 0.95 |  |
|  |  | Methionine | 0.042 | 0.69 | 0.61 |  |
|  |  | Leucine | -0.061 | 0.87 | 0.86 |  |
|  |  | Histidine | 0.145 | 0.74 | 0.61 |  |
|  |  | Arachidonic Acid | -0.0011 | 0.62 | 1.0 |  |
|  |  | Vitamin B12 | 0.053 | 0.67 | 0.97 |  |
|  |  | Choline | 0.073 | 0.80 | 0.76 |  |
|  |  | Zinc | -0.049 | 0.63 | 0.63 |  |
|  |  | Magnesium | -0.078 | 0.84 | 0.86 |  |
|  |  |  |  |  |  |  |
| Methionine | 0.0037 | Cereals | 0.00055 | 0.63 | 1.0 | 0.053 (2.4%) |
|  |  | Pulses | -0.010 | 0.98 | 1.0 |  |
|  |  | Non-Ruminant Meat | 0.00076 | 0.76 | 1.0 |  |
|  |  | Tryptophan | -0.14 | 0.67 | 0.93 |  |
|  |  | Isoleucine | 0.85 | 0.92 | 0.95 |  |
|  |  | DHA+EPA | -0.042 | 0.64 | 0.86 |  |
|  |  | Vitamin D | 0.13 | 0.78 | 0.93 |  |
|  |  | Niacin | -0.0092 | 0.84 | 0.54 |  |
|  |  |  |  |  |  |  |
| Phenylalanine | 0.028 | Cereals | 0.00071 | 0.83 | 1.0 | 0.081 (4.1%) |
|  |  | Roots & Tubers | -0.00017 | 0.65 | 1.0 |  |
|  |  | Ruminant Meat | 0.0013 | 0.76 | 1.0 |  |
|  |  | Non-Ruminant Meat | -0.00018 | 0.86 | 1.0 |  |
|  |  | Tryptophan | 0.54 | 0.99 | 0.83 |  |
|  |  |  |  |  |  |  |
| Threonine | 0.048 | Cereals | 0.000035 | 0.78 | 1.0 | 0.71 (2.6%) |
|  |  | Pulses | 0.0085 | 0.72 | 1.0 |  |
|  |  | Nuts | 0.0040 | 0.69 | 1.0 |  |
|  |  | Ruminant Meat | 0.0062 | 0.78 | 1.0 |  |
|  |  | Non-Ruminant meat | 0.0049 | 0.86 | 1.0 |  |
|  |  | Milk | 0.00049 | 0.67 | 1.0 |  |
|  |  | Tryptophan | -0.15 | 0.66 | 0.55 |  |
|  |  | Phenylalanine | 1.26 | 0.70 | 0.60 |  |
|  |  |  |  |  |  | *(Continued)* |
| **Supplementary Table S1.** Continued | | |  |  |  |  |
| Response | Intercept | Variable | Value | Strength | Direction | Residual SE |
| Tryptophan | 0.13 | Cereals | 0.011 | 0.65 | 1.0 |  |
|  |  | Sweeteners | 0.00046 | 0.65 | 1.0 |  |
|  |  | Non-Ruminant Meat | 0.0095 | 0.72 | 1.0 |  |
|  |  | Vitamin B12 | 0.048 | 0.72 | 0.94 |  |
|  |  |  |  |  |  |  |
| Valine | 0.011 | Cereals | 0.00054 | 0.70 | 1.0 |  |
|  |  | Pulses | -0.0030 | 0.65 | 1.0 |  |
|  |  | Roots & Tubers | 0.00022 | 0.62 | 1.0 |  |
|  |  | Milk | 0.00035 | 0.98 | 1.0 |  |
|  |  | Eggs | 0.0019 | 0.77 | 1.0 |  |
|  |  | Threonine | 0.31 | 0.74 | 0.76 |  |
|  |  | Phenylalanine | 0.31 | 0.73 | 0.79 |  |
|  |  | Isoleucine | 0.45 | 0.97 | 0.78 |  |
|  |  |  |  |  |  |  |
| Alpha Linolenic Acid | 0.43 | Oilseeds | 0.16 | 0.96 | 1.0 | 2.3 (10.5%) |
|  |  | Berries | 0.84 | 0.82 | 1.0 |  |
|  |  | Water | -338,044 | 0.68 | 0.99 |  |
|  |  |  |  |  |  |  |
| Arachidonic Acid | -0.56 | Oilseeds | 0.10 | 0.80 | 1.0 | 6.43 (29.5%) |
|  |  | Non=Ruminant Meat | 0.39 | 1.0 | 1.0 |  |
|  |  | Milk | 0.0091 | 0.81 | 1.0 |  |
|  |  |  |  |  |  |  |
| DHA+EPA | -0.0057 | Eggs | 0.0019 | 1.0 | 1.0 | 0.061 (32.0%) |
|  |  | Vitamin B12 | 0.0017 | 0.79 | 0.58 |  |
|  |  | Water | -29,322 | 0.62 | 0.91 |  |
|  |  |  |  |  |  |  |
| Linoleic Acid | -0.16 | Nuts | 0.023 | 0.98 | 1.0 | 0.178 (9.0%) |
|  |  | Oilseeds | 0.0051 | 0.95 | 1.0 |  |
|  |  | Non-Ruminant Meat | 0.0089 | 0.85 | 1.0 |  |
|  |  | Alpha Linolenic Acid | 0.15 | 0.97 | 0.86 |  |
|  |  | Vitamin E | 0.16 | 0.81 | 0.60 |  |
|  |  | Choline | 0.61 | 0.67 | 0.85 |  |
|  |  | Selenium | -0.23 | 0.87 | 0.80 |  |
|  |  | Potassium | -0.72 | 0.61 | 0.88 |  |
|  |  | Magnesium | 0.79 | 0.87 | 0.90 |  |
|  |  |  |  |  |  |  |
| Choline | -0.097 | Roots & Tubers | 0.0012 | 1.0 | 1.0 | 0.130 (17.0%) |
|  |  | Milk | -0.000029 | 0.68 | 1.0 |  |
|  |  | Eggs | 0.0088 | 1.0 | 1.0 |  |
|  |  | Valine | 0.16 | 0.86 | 0.55 |  |
|  |  | Phosphorus | 0.073 | 0.62 | 0.58 |  |
|  |  |  |  |  |  |  |
| Folate | -0.47 | Vitamin C | 0.39 | 0.68 | 0.58 | 1.03 (56.1%) |
|  |  | Magnesium | 0.72 | 0.71 | 0.62 |  |
|  |  |  |  |  |  | *(Continued)* |
| **Supplementary Table S1.** Continued | | |  |  |  |  |
| Response | Intercept | Variable | Value | Strength | Direction | Residual SE |
| Niacin | 0.0051 | Cereals | 0.0050 | 0.95 | 1.0 | 0.204 (8.7%) |
|  |  | Pulses | -0.013 | 0.64 | 1.0 |  |
|  |  | Nuts | 0.011 | 0.64 | 1.0 |  |
|  |  | Protein | 1.12 | 0.73 | 0.80 |  |
|  |  | Phenylalanine | -0.74 | 0.68 | 0.90 |  |
|  |  | Cysteine | 0.35 | 0.67 | 0.71 |  |
|  |  | Phosphorus | -0.39 | 0.66 | 0.69 |  |
|  |  |  |  |  |  |  |
| Riboflavin | 0.028 | Milk | 0.0020 | 0.97 | 1.0 | 0.083 (3.7%) |
|  |  | Histidine | 0.62 | 0.93 | 0.75 |  |
|  |  | EHA+EPA | -0.92 | 0.87 | 0.95 |  |
|  |  | Niacin | -0.45 | 0.68 | 0.58 |  |
|  |  | Choline | 1.1 | 0.99 | 0.82 |  |
|  |  | Magnesium | -0.035 | 0.69 | 0.72 |  |
|  |  |  |  |  |  |  |
| Thiamin | 0.062 | Cereals | 0.0048 | 0.73 | 1.0 | 0.222 (6.9%) |
|  |  | Nuts | 0.015 | 0.72 | 1.0 |  |
|  |  | Roots & Tubers | 0.0015 | 0.72 | 1.0 |  |
|  |  | Valine | 0.14 | 0.74 | 0.57 |  |
|  |  | Cysteine | 0.12 | 0.62 | 0.91 |  |
|  |  | DHA+EPA | -3.11 | 0.94 | 0.96 |  |
|  |  | Arachidonic Acid | 0.027 | 0.92 | 0.95 |  |
|  |  | Vitamin E | 0.105 | 0.66 | 0.81 |  |
|  |  | Folate | 0.35 | 0.99 | 0.51 |  |
|  |  | Phosphorus | 0.18 | 0.61 | 0.74 |  |
|  |  |  |  |  |  |  |
| Vitamin A | 0.45 | Roots & Tubers | 0.0063 | 0.92 | 1.0 | 0.466 (46.0%) |
|  |  | Milk | 0.0019 | 0.64 | 1.0 |  |
|  |  |  |  |  |  |  |
| Vitamin B12 | 0.0035 | Berries | -0.077 | 0.63 | 1.0 | 0.218 (8.8%) |
|  |  | Ruminant Meat | 0.034 | 0.99 | 1.0 |  |
|  |  | Non-Ruminant Meat | 0.015 | 1.00 | 1.0 |  |
|  |  | Milk | 0.0056 | 0.99 | 1.0 |  |
|  |  | Eggs | 0.035 | 0.76 | 1.0 |  |
|  |  | Arachidonic Acid | -0.015 | 0.80 | 0.57 |  |
|  |  |  |  |  |  |  |
| Vitamin B6 | 0.094 | Cereals | 0.010 | 0.77 | 1.0 | 0.75 (29.0%) |
|  |  | Nuts | 0.037 | 0.80 | 1.0 |  |
|  |  | Vegetables | -0.0019 | 0.68 | 1.0 |  |
|  |  | Fruits | 0.0040 | 0.66 | 1.0 |  |
|  |  | Vitamin A | 0.62 | 0.66 | 0.91 |  |
|  |  |  |  |  |  |  |
| Vitamin C | 0.402 | Vegetables | 0.0048 | 0.84 | 1.0 | 0.379 (33.0%) |
|  |  | Fruits | 0.0056 | 0.99 | 1.0 |  |
|  |  | Roots & Tubers | 0.0095 | 1.00 | 1.0 |  |
|  |  |  |  |  |  |  |
| Vitamin D | 0.124 |  |  |  |  | 0.144 (116%) |
|  |  |  |  |  |  | *(Continued)* |
| **Supplementary Table S1.** Continued | | |  |  |  |  |
| Response | Intercept | Variable | Value | Strength | Direction | Residual SE |
| Vitamin E | -0.163 | Nuts | 0.064 | 0.96 | 1.0 | 0.313 (29.5%) |
|  |  | Oilseeds | 0.031 | 0.99 | 1.0 |  |
|  |  | Selenium | 0.069 | 0.64 | 0.65 |  |
|  |  |  |  |  |  |  |
| Vitamin K | 0.044 | Pulses | 0.011 | 0.96 | 1.0 | 0.181 (21.8%) |
|  |  | Vegetables | 0.0077 | 1.00 | 1.0 |  |
|  |  | Alpha Linolenic Acid | 0.108 | 0.99 | 0.72 |  |
|  |  |  |  |  |  |  |
| Calcium | -0.016 | Sweeteners | 0.0018 | 0.91 | 1.0 | 0.076 (8.9%) |
|  |  | Vegetables | -0.00030 | 0.73 | 1.0 |  |
|  |  | Milk | 0.0022 | 1.00 | 1.0 |  |
|  |  | Riboflavin | 0.109 | 0.63 | 0.60 |  |
|  |  | Iron | 0.092 | 0.64 | 0.81 |  |
|  |  |  |  |  |  |  |
| Copper | 0.087 | Pulses | 0.027 | 0.61 | 1.0 | 0.318 (7.6%) |
|  |  | Nuts | 0.028 | 0.92 | 1.0 |  |
|  |  | Fruits | 0.0047 | 0.76 | 1.0 |  |
|  |  | Roots & Tubers | 0.0027 | 0.74 | 1.0 |  |
|  |  | Spices | 0.0049 | 0.91 | 1.0 |  |
|  |  | Vitamin B6 | -0.12 | 0.67 | 0.69 |  |
|  |  | Magnesium | 1.19 | 0.96 | 0.66 |  |
|  |  |  |  |  |  |  |
| Iron | -0.023 | Cereals | 0.0069 | 0.97 | 1.0 | 0.200 (7.3%) |
|  |  | Sweeteners | 0.00090 | 0.83 | 1.0 |  |
|  |  | Pulses | 0.026 | 0.73 | 1.0 |  |
|  |  | Nuts | -0.0046 | 0.68 | 1.0 |  |
|  |  | Fruits | 0.0018 | 0.74 | 1.0 |  |
|  |  | Spices | 0.0041 | 0.71 | 1.0 |  |
|  |  | Ruminant Meat | 0.0026 | 0.74 | 1.0 |  |
|  |  | Zinc | 0.014 | 0.61 | 0.61 |  |
|  |  | Magnesium | -0.41 | 0.63 | 0.77 |  |
|  |  | Copper | 0.41 | 0.99 | 0.55 |  |
|  |  |  |  |  |  |  |
| Magnesium | -0.058 | Cereals | -0.0019 | 0.70 | 1.0 | 0.381 (14.8%) |
|  |  | Ruminant meat | -0.0020 | 0.64 | 1.0 |  |
|  |  | Non-Ruminant Meat | -0.0011 | 0.62 | 1.0 |  |
|  |  | Leucine | 0.797 | 0.75 | 0.70 |  |
|  |  | Vitamin B6 | 0.697 | 0.92 | 0.52 |  |
|  |  |  |  |  |  |  |
| Phosphorus | -0.28 | Cereals | 0.0020 | 0.81 | 1.0 | 0.177 (5.3%) |
|  |  | Sweeteners | -0.00014 | 0.70 | 1.0 |  |
|  |  | Vegetables | 0.0019 | 0.71 | 1.0 |  |
|  |  | Ruminant Meat | -0.0044 | 0.78 | 1.0 |  |
|  |  | Milk | 0.0018 | 0.87 | 1.0 |  |
|  |  | Protein | 0.534 | 0.85 | 0.87 |  |
|  |  | DHA+EPA | 1.69 | 0.81 | 0.95 |  |
|  |  | Folate | -0.057 | 0.86 | 0.54 |  |
|  |  | Magnesium | 0.55 | 0.63 | 0.88 |  |
|  |  |  |  |  |  | *(Continued)* |
|  |  |  |  |  |  |  |
| **Supplementary Table S1.** Continued | | |  |  |  |  |
| Response | Intercept | Variable | Value | Strength | Direction | Residual SE |
| Potassium | 0.021 | Sweeteners | 0.00015 | 0.67 | 1.0 | 0.081 (7.9%) |
|  |  | Fruits | 0.0014 | 0.99 | 1.0 |  |
|  |  | Roots & Tubers | 0.0018 | 0.99 | 1.0 |  |
|  |  | Methionine | 0.212 | 0.68 | 0.57 |  |
|  |  | Vitamin B6 | -0.079 | 0.63 | 0.76 |  |
|  |  | Vitamin A | 0.050 | 0.97 | 0.93 |  |
|  |  | Niacin | -0.122 | 0.98 | 0.89 |  |
|  |  | Phosphorus | 0.107 | 0.63 | 0.75 |  |
|  |  | Copper | 0.108 | 0.62 | 0.73 |  |
|  |  |  |  |  |  |  |
|  |  |  |  |  |  |  |
| Selenium | -0.26 | Roots & Tubers | -0.0061 | 0.78 | 1.0 | 0.456 (10.8%) |
|  |  | Valine | 0.44 | 0.66 | 0.59 |  |
|  |  | Threonine | -3.53 | 0.75 | 0.72 |  |
|  |  | Leucine | -1.53 | 0.90 | 0.75 |  |
|  |  | Histidine | 3.34 | 0.82 | 0.55 |  |
|  |  | Niacin | 1.37 | 0.79 | 0.86 |  |
|  |  | Folate | 0.012 | 0.87 | 0.57 |  |
|  |  | Choline | 2.04 | 0.67 | 0.61 |  |
|  |  | Phosphorus | 0.72 | 0.66 | 0.78 |  |
|  |  |  |  |  |  |  |
| Zinc | -0.075 | Cereals | 0.0014 | 0.71 | 1.0 | 0.106 (4.6%) |
|  |  | Sweeteners | 0.000042 | 0.81 | 1.0 |  |
|  |  | Ruminant Meat | 0.0046 | 0.91 | 1.0 |  |
|  |  | Protein | 0.58 | 0.84 | 0.83 |  |
|  |  | Valine | 0.71 | 0.81 | 0.57 |  |
|  |  | Isoleucine | -0.68 | 0.69 | 0.85 |  |
|  |  | Choline | 0.0309 | 0.63 | 0.54 |  |
|  |  | Copper | 0.11 | 0.74 | 0.59 |  |
|  |  |  |  |  |  |  |
| Fruits | 26.2 | Vegetables | 0.396 | 0.86 | 0.72 | 27.3 (71%) |
|  |  |  |  |  |  |  |
| Vegetables | 19.7 | Cereals | 0.079 | 0.68 | 0.62 | 21.7 (70.3%) |
|  |  |  |  |  |  |  |
| Oilseeds | 13.1 |  |  |  |  | 27.7 (211%) |
|  |  |  |  |  |  |  |
| Cereals | 115.7 | Oilseeds | 1.93 | 0.66 | 0.62 | 87.7 (62.2%) |
|  |  |  |  |  |  |  |
| Sweeteners | 175.5 |  |  |  |  | 372.1 (212%) |
|  |  |  |  |  |  |  |
| Roots & Tubers | 40.3 |  |  |  |  | 66.6 (165%) |
|  |  |  |  |  |  |  |
| Pulses | -2.62 | Eggs | 2.81 | 0.77 | 0.66 | 59.8 (279%) |
|  |  |  |  |  |  |  |
| Nuts | 8.27 |  |  |  |  | 19.6 (204%) |
|  |  |  |  |  |  |  |
| Ruminant Meat | 24.8 |  |  |  |  | 38.9 (157%) |
|  |  |  |  |  |  |  |
| Non-Ruminant Meat | 31.7 | Oilseeds | 1.41 | 0.98 | 0.78 | 45.2 (90%) |
|  |  |  |  |  |  | *(Continued)* |
| **Supplementary Table S1.** Continued | | |  |  |  |  |
| Response | Intercept | Variable | Value | Strength | Direction | Residual SE |
| Milk | -111.7 | Pulses | -1.3 | 0.61 | 0.95 | 200.2 (115%) |
|  |  | Ruminant Meat | 8.12 | 0.99 | 0.67 |  |
|  |  | Non-Ruminant Meat | 0.80 | 0.62 | 0.88 |  |
|  |  | Eggs | 8.33 | 0.75 | 0.80 |  |
|  |  |  |  |  |  |  |
| Eggs | 2.95 | Vegetables | 0.087 | 0.88 | 0.55 | 4.79 (55%) |
|  |  | Non-Ruminant Meat | 0.058 | 0.94 | 0.87 |  |
|  |  |  |  |  |  |  |
| Spices | 3.29 | Oilseeds | 0.19 | 0.81 | 0.86 | 4.36 (105%) |
|  |  | Berries | -1.64 | 0.77 | 0.70 |  |
|  |  |  |  |  |  |  |
| CO2Eq | 0.0000988 | Berries | -0.000138 | 0.77 | 1.0 | 0.00020 (20%) |
|  |  | Ruminant Meat | 0.00000703 | 0.70 | 1.0 |  |
|  |  | N2O | 0.054 | 0.76 | 1.0 |  |
|  |  |  |  |  |  |  |
| CH4 | 0.0000047 | Berries | -0.0000066 | 0.77 | 1.0 | 0.0000033 (13%) |
|  |  | Ruminant Meat | 0.000000335 | 0.85 | 1.0 |  |
|  |  | N2O | 10.7 | 1.0 | 0.83 |  |
|  |  |  |  |  |  |  |
| N2O | 0.000000597 | Ruminant Meat | 0.000000036 | 0.89 | 1.0 | 0.0000013 (89%) |
|  |  |  |  |  |  |  |
| Water | 0.000000213 | Vegetables | 0.0000000039 | 0.63 | 1.0 | 0.00000063 (158%) |
